# Analysis of Classical Time-Trial Performance and Technique-Specific Physiological Determinants in Elite Female Cross-Country Skiers

**DOI:** 10.3389/fphys.2016.00326

**Published:** 2016-08-03

**Authors:** Øyvind Sandbakk, Thomas Losnegard, Øyvind Skattebo, Ann M. Hegge, Espen Tønnessen, Jan Kocbach

**Affiliations:** ^1^Department of Neuroscience, Centre for Elite Sports Research, Norwegian University of Science and TechnologyTrondheim, Norway; ^2^Department of Physical Performance, Norwegian School of Sports SciencesOslo, Norway; ^3^The Norwegian Olympic FederationOslo, Norway

**Keywords:** aerobic capacity, cross-country skiing, endurance sport, work economy, women

## Abstract

The present study investigated the contribution of performance on uphill, flat, and downhill sections to overall performance in an international 10-km classical time-trial in elite female cross-country skiers, as well as the relationships between performance on snow and laboratory-measured physiological variables in the double poling (DP) and diagonal (DIA) techniques. Ten elite female cross-country skiers were continuously measured by a global positioning system device during an international 10-km cross-country skiing time-trial in the classical technique. One month prior to the race, all skiers performed a 5-min submaximal and 3-min self-paced performance test while roller skiing on a treadmill, both in the DP and DIA techniques. The time spent on uphill (*r* = 0.98) and flat (*r* = 0.91) sections of the race correlated most strongly with the overall 10-km performance (both *p* < 0.05). Approximately 56% of the racing time was spent uphill, and stepwise multiple regression revealed that uphill time explained 95.5% of the variance in overall performance (*p* < 0.001). Distance covered during the 3-min roller-skiing test and body-mass normalized peak oxygen uptake (VO_2peak_) in both techniques showed the strongest correlations with overall time-trial performance (*r* = 0.66–0.78), with DP capacity tending to have greatest impact on the flat and DIA capacity on uphill terrain (all *p* < 0.05). Our present findings reveal that the time spent uphill most strongly determine classical time-trial performance, and that the major portion of the performance differences among elite female cross-country skiers can be explained by variations in technique-specific aerobic power.

## Introduction

Cross-country skiing is one of the most demanding endurance sports and involves whole body exercise of varying techniques, intensity and duration. The race courses consist of approximately one-third uphill, one-third flat, and one-third downhill and since ~50% of the total time is spent skiing uphill, uphill performance is regarded as the major determinant of success (Norman and Komi, 1987; Bergh and Forsberg, 1992; Mognoni et al., 2001; Sandbakk et al., 2011; Bolger et al., 2015). During time-trial competitions, skiers utilize a positive pacing strategy (i.e., a general reduction in speed throughout the race), with increased exercise intensity on uphill and reduced effort on downhill sections (Sandbakk et al., 2011; Bolger et al., 2015; Losnegard et al., 2016). In addition to increasing their physiological effort in uphill terrain, skiers are able to produce greater work rates at a given metabolic intensity (i.e., higher efficiency) compared to flat and downhill terrain (Sandbakk et al., 2013). This provides a unique advantage for employing a terrain-variable pacing in cross-country skiing and further highlights uphill terrain as a main determinant of overall performance.

Competing at an international level in cross-country skiing requires an exceptionally high aerobic power, and world-class cross-country skiers exhibit maximal oxygen uptake (VO_2max_) values of >80 and >70 mL·min^−1^·kg^−1^ for men and women, respectively (Saltin and Astrand, 1967; Ingjer, 1991; Holmberg et al., 2007; Tonnessen et al., 2015; Sandbakk et al., 2016). While VO_2max_ is consistently attained using diagonal stride skiing (DIA) in uphill terrain or during running, peak oxygen uptake (VO_2peak_) of elite skiers employing sub-techniques used in flat terrain, such as double poling (DP), are reported to be considerably lower (Holmberg et al., 2007; Losnegard and Hallen, 2014; Sandbakk et al., 2016). However, the ability to utilize high aerobic power while performing these various techniques is crucial for performance (Sandbakk and Holmberg, 2014; Holmberg, 2015). In a recent study we demonstrated that world-class female skiers perform better and exhibit higher VO_2peak_ than their national level counterparts, both in DIA on uphill and DP on nearly flat terrain (Sandbakk et al., 2016). In addition, the technical complexity of cross-country skiing may augment performance level variations also in the ability to efficiently convert energy into power and speed (i.e., work economy or efficiency) in the different sub-techniques. Performance level differences in efficiency has recently been shown in roller ski skating both for male and female skiers (Sandbakk et al., 2010, 2013; Ainegren et al., 2013), and gross efficiency while skating in the laboratory was significantly correlated with performance in the same technique and terrain on snow (Sandbakk et al., 2011). However, in classical roller skiing the efficiency differences between performance levels seem less pronounced (Sandbakk et al., 2016) and the relationships to performance on snow has not yet been examined.

Although the physiological and biomechanical characteristics of male cross-country skiers have been analyzed in detail in the laboratory in recent decades (Sandbakk and Holmberg, 2014), there is a particular lack of literature regarding the sport-specific demands faced by elite female skiers. Therefore, the present study investigated the contribution of performance on uphill, flat, and downhill sections to overall performance in an international 10-km classical time-trial in elite female cross-country skiers, as well as the relationships between performance on snow and physiological variables in the DP and DIA techniques. Our major hypotheses were that uphill terrain is the main performance differentiating terrain, and that high VO_2peak_ in both DP and DIA are primary laboratory determinants of time-trial performance.

## Methods

### Participants

Ten elite female Norwegian cross-country skiers who ranged from the highest ranked skier in the world to being among the top 15 in Norwegian cup races (Fis, 2015) participated in the study (Table 1). All athletes were healthy and free of injuries at the time of testing, and the study was approved by the Regional Committee of Medical and Health Research Ethics in Central Norway and conducted in accordance with the Declaration of Helsinki. Prior to starting the study, all participants signed an informed consent form and were made aware that they could withdraw at any point without providing an explanation.

**Table 1:** **Anthropometric, physiological, and performance characteristics of the 10 elite female cross-country skiers involved in this study (mean ± *SD*)**.

**Variables**	
Age (year)	24.5 ± 3.9
Body height (cm)	168 ± 4
Body mass (kg)	61.9 ± 5.6
Body mass index (kg·m^−2^)	21.8 ± 1.3
Distance FIS points	44.6 ± 34.8
VO_2max_ (L·min^−1^)	4.20 ± 0.28
VO_2max_ (mL·min^−1^·kg^−1^)	68.0 ± 4.8
Training (hours·year^−1^)	818 ± 78

*FIS, the International Ski Federation; VO_2max_, maximal oxygen uptake, highest value measured last 6 months before the study was conducted*.

### Overall design

**Figure 1:**
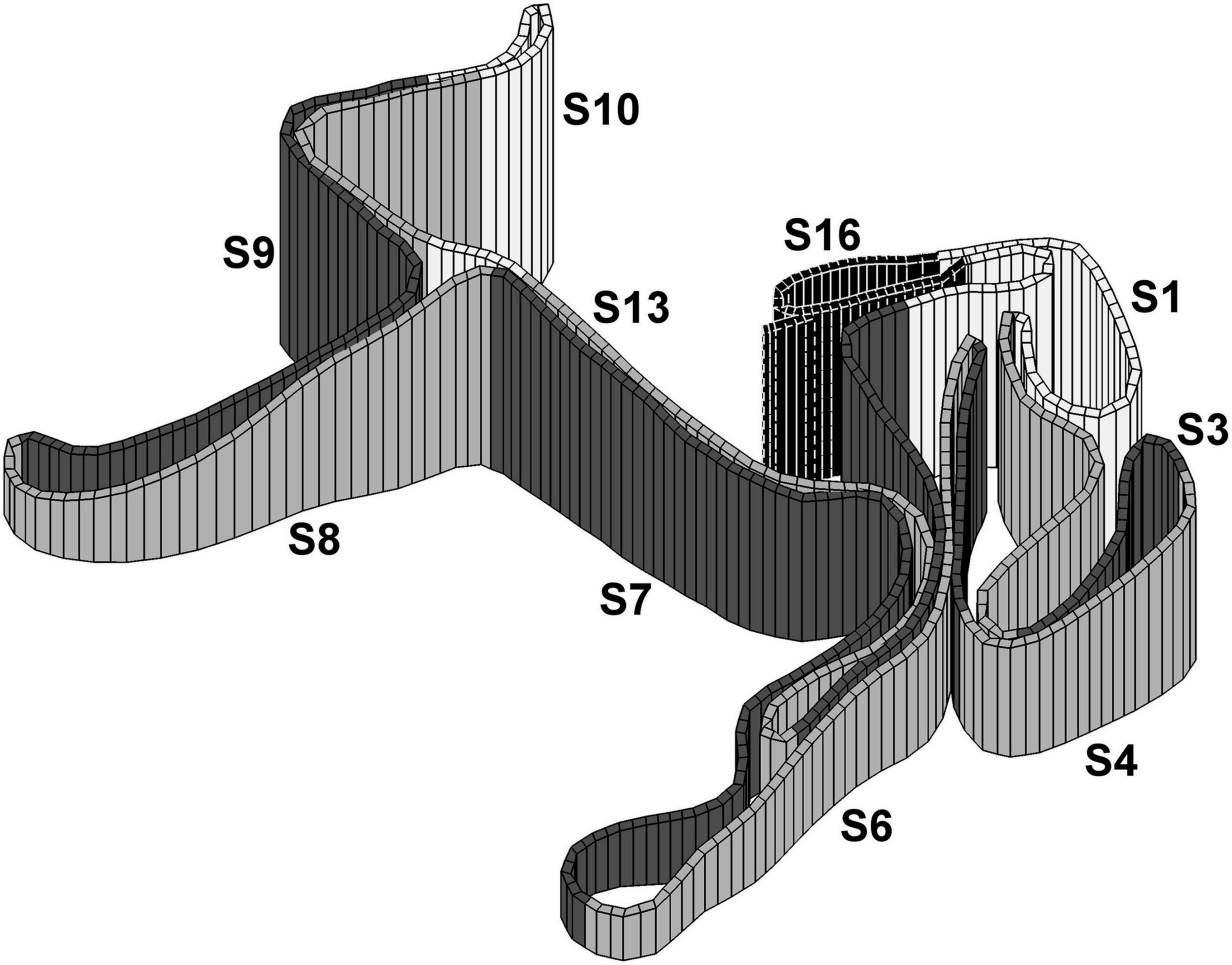
**3-dimensional illustration of the 16 sections (S1–16) of the 5-km course examined in the current study**. Note that each section is included in both laps except S16 which contains in total 435 m of flat sections that differ between the two laps (i.e., start and finish section).

In mid-November, all skiers were tracked by a global positioning system (GPS) device during an international 10-km cross-country skiing International Ski Federation (FIS) regulated competition in the classical technique with an individual time-trial format. The race was performed on a 5-km competition course which was mapped with a coupled GPS and barometer in an inertial navigation system (INS) to provide a valid course and elevation profile (Figure 1). The skiers' data were adapted to the standard racecourse for analysis of time and speed for pre-defined uphill, flat, and downhill sections. One month prior to the competition, all skiers completed exercise and performance testing in the laboratory. These tests consisted of (1) two 5-min submaximal stages to measure exercise economy, one using DP and one using DIA, at a constant workload, and (2) two 3-min self-paced performance tests to measure treadmill performance and peak physiological responses, one using DP and one using DIA. In these tests, speed and cardiorespiratory variables were monitored continuously, and blood lactate concentration was analyzed after each test. During the 3-min tests, the total distance covered (as a measure of performance) and peak oxygen uptake (VO_2peak_) were determined.

### Competition analysis

Ski equipment was individualized to the specific athlete's racing preferences, including pole length (82 ± 2% of body height), boot style, ski length, ski base material, and camber stiffness. All ski base preparations, including grinds, structure, and waxing, were individualized for the prevailing conditions by the Norwegian cross-country skiing wax team. The weather conditions for the race day were stable throughout the whole competition: no wind, partly cloudy, −3°C, and ~93% humidity. The course was covered with hard packed mixed snow and machine-prepared on the evening prior to the competition. The course was set in an open area with minimal tree cover and no mountains to interfere with GPS signals. Course and elevation profiles were standardized using a Garmin Forerunner GPS (Garmin Ltd., Olathe, Kansas, USA) that collected position data at a sampling rate of 1 Hz coupled with an Apertus INS (Apertus Skiing Sensor, Apertus AS, Asker, Norway) with integrated barometry that collected accurate elevation data. The course profile was divided into uphill, flat, and downhill sections that made up 41, 23, and 36% of the total competition (i.e., the exact course was 9.8 km, but will hereafter be named 10 km), respectively (see Table 2 for an overview of the different sections). As 435 m of flat terrain was part of the start and finish of the race, these sections were not included in the comparisons of the two identical 4.7 km laps. A section boundary was defined at each point where there was a change between positive and negative gradient in the course profile. The uphill and downhill sections were characterized by a minimum elevation difference of 10 m within the section. A section with an ascent or decent of < 10 m was defined as a flat section. Adjacent flat sections were merged into longer sections that in some cases contained small uphill and downhill parts.

**Table 2:** **Section length and elevation, as well as the time spent, mean speed and coefficient of variation (CV) averaged over both laps of the 10-km time trial for 10 elite female cross-country skiers**.

**Terrain**	**Track section**	**Section length (m)**	**Elevation (m/%)**	**Mean section time (s)**	**Mean speed in section**	
					**Speed (m·s^−1^)**	**CV (%)**
Uphill	S3	195	11/6	42 ± 2	4.7	5.5
	S5	170	13/8	41 ± 3	4.2	6.3
	S7	675	36/5	162 ± 10	4.2	6.4
	S9	500	44/9	152 ± 11	3.3	7.6
	S14	335	23/7	85 ± 4	4.0	5.2
	Total	1875	127/7	483 ± 31	4.1	6.2
Flat	S1^#^	305	0/0	46 ± 3	6.7	6.5
	S10	180	0/0	33 ± 2	5.4	4.9
	S12	70	1/2	10 ± 1	6.8	8.0
	S15	175	1/1	29 ± 1	6.2	4.3
	S16^¤^	435S16^¤^	0/0	75 ± 3	5.8	3.7
	Total	1165	3/0	193 ± 10	6.2	5.5
Downhill	S2	285	14/5	32 ± 1	8.8	4.0
	S4	210	11/5	25 ± 1	8.5	3.4
	S6	480	28/6	46 ± 2	10.4	3.4
	S8	365	35/10	31 ± 2	11.9	5.4
	S11	230	11/5	30 ± 1	7.8	4.1
	S13	510	26/5	55 ± 2	9.4	3.1
	Total	2080	125/6	218 ± 8	9.5	3.9

# *Initial speed of zero at first lap*;

¤ *Contains all the flat sections that differ between the two laps due to the sections from start and toward finish; all other sections (S1–15) were performed twice so the exact race distance was 9.805 km*.

During the competition, each participant wore a Garmin Forerunner GPS that collected position data and heart rate data at a sampling rate of 1 Hz. The GPS watches were turned on at least 30 min before the start of the race to ensure proper GPS fixing in order to minimize inaccuracy in GPS data. To further reduce inaccuracy, the recorded position data of each participant was projected onto the standard course. A projection algorithm assigns a virtual split time for each participant and lap to predefined points on the standard course with spacing of ~10 m. The virtual split times are calculated using a combination of proximities including the skiers' GPS course, the standard course, and distance traveled since the previous identified virtual split time. The average projection distance from the GPS course for each participant to the corresponding point on the standard course varied between 3 and 5.5 m for all participants and loops. The time each participant spent in a section was calculated based on virtual split times. Speed for each section was calculated by dividing the length of a section by the time elapsed within that section and given as meter per second (m·s^−1^).

### Laboratory testing

Roller ski tests were performed on a 3-by-4.5-m motor-driven roller ski treadmill (Rodby, Södertalje, Sweden). Incline and speed were calibrated before, during and after the study. The treadmill belt was covered with non-slip rubber that allowed the skiers to use their own poles (length: 83 ± 2% of body height), equipped with carbide tips. Participants wore a safety harness connected to an automatic emergency brake throughout the tests. The speed during the self-paced 3-min tests was controlled using two laser beams (BDL120, Black and Decker, Towson, Maryland, USA) separated by 60 cm.

All skiers wore their own classical cross-country skiing shoes, but used the same pair of Swenor roller skis (Swenor, Sarpsborg, Norway) with an NNN binding system (Rottefella, Klokkarstua, Norway) and standard wheels. The rolling friction coefficient (μ) was tested before, at various times during, and after the study using the towing test described by Sandbakk et al. (2010), providing an average μ-value of 0.028.

Respiratory variables were measured using open-circuit indirect calorimetry. Expired gas was passed through a mixing chamber and analyzed continuously (Oxycon Pro, Jaeger GmbH, Hoechberg, Germany). The instruments were calibrated against ambient air and commercial gas of known concentrations of O_2_ (14.90%) and CO_2_ (6.05%) before the start of each test day. The flow transducer (Triple V, Erich Jaeger GmbH, Hoechberg, Germany) was calibrated using a 3-L high-precision calibration syringe (5530 series, Hans Rudolph Inc., Kansas City, Missouri, USA). Heart rate was continuously measured with a Polar S610i monitor (Polar Electro Oy, Kempele, Finland) and synchronized with the Oxycon Pro measurement system. Blood lactate concentration in 5 μL of blood taken from the fingertip was measured using the Lactate Pro LT-1710*t* kit (ArkRay Inc, Kyoto, Japan), validated by Medbo et al. (2000). Rating of perceived exertion (RPE) was recorded using the 6–20 point Borg Scale (Borg, 1982). The participants' body-mass was determined before each test with an electronic body-mass scale (Seca model nr: 877; Seca GmbH & Co., Hamburg, Germany).

Following 15–20 min of low-to-moderate intensity warm-up on the treadmill, alternating between the DP and DIA techniques, each skier performed two 5-min stages of roller skiing at a constant speed both in DP (at an incline of 3% and 3.5 m·s^−1^) and DIA (12% and 2 m·s^−1^). The same order was used by all participants. The inclines used in DP and DIA are based on previous research and represent typical inclines where these techniques are employed by elite skiers (Pellegrini et al., 2013; Stoggl and Holmberg, 2016). The corresponding speeds for each technique were chosen to obtain similar RPE and blood lactate concentration across techniques. This was based on previous testing of elite cross-country skiers of a similar performance level in our laboratory. A 2-min recovery was given between the two submaximal stages. Cardiorespiratory variables were monitored continuously and the averages for the final 2 min provided the steady-state values utilized for further analyses. Blood lactate and RPE were assessed immediately after both submaximal stages.

A 10-min recovery period followed the submaximal tests, before each skier first carried out a 3-min self-paced DP test to failure at 3% incline and, following a 20-min break, the DIA test at 12% incline. The rationale for using this protocol is based on our previous experience that a 3-min test provides a reliable performance measure and is well-suited to detect VO_2peak_ (Losnegard et al., 2012). The speed was fixed for the first 30 s to avoid over-pacing (5 and 3 m·s^−1^ for the DP and DIA tests, respectively). Thereafter, the skiers controlled the speed by adjusting their position on the treadmill relative to the laser beams. Each contact of the front wheels with the front or the rear laser resulted in an increase or reduction in speed, respectively, by 0.25–0.5 m·s^−1^ in the case of DP and 0.25 m·s^−1^ for DIA. Each skier received continuous visual and verbal feedback concerning the time that had elapsed, but were blinded to the performance of the other skiers. Changes in speed and the accumulated distance skied were registered automatically. Cardiorespiratory variables were monitored continuously and the highest average VO_2_ during a continuous 30-s period was defined as VO_2peak_. The highest 5-s heart rate was defined as peak heart rate. Fingertip blood samples were collected for determination of blood lactate concentration, and RPE was recorded ~1.5 min after each test.

### FIS points

The performance level on snow was based on the FIS point system (Fis, 2015). According to FIS, a skier's rank is set relative to a zero-point standard established by the top-ranked skier in the world, where better skiers have fewer FIS points. A skier's total score for a given race is calculated by adding race points determined by a comparison between the individual's ski time with the winner's time, and by adding racing penalties.

### Statistical analysis

All data were checked for normality using the Shapiro-Wilk test. In cases where data were not normally distributed, a non-parametric test was used. The coefficient of variation in race speed between the athletes (standard deviation/mean) was calculated within each terrain section of the race and presented as a percentage. Correlations between overall performance and speed in each uphill, flat, and downhill section were estimated with Pearson's product–moment correlation coefficient test, or the non-parametric Spearman's rank correlation. To compare if a correlation is statistically larger than others, a *t*-test for independent correlations according to Chen and Popovich (2002) was applied. In addition, a stepwise multiple regression model was constructed, with the total race time as the dependent variable (DV) and the time spent uphill, flat, and downhill as independent variables (IVs). The following variables were extracted from the multiple regression analysis: (1) the coefficient of determination change when an IV is added to the model (*R*^2^ change); (2) the standardized beta coefficient (std. β); (3) the semi partial *R*^2^ (the decrease in *R*^2^ when an IV is removed from the final model) and (4) *p*-values. All statistical analyses were processed using SPSS 11.0 Software for Windows (SPSS Inc., Chicago, IL). Statistical significance was set at an alpha level of < 0.05.

## Results

**Figure 2:**
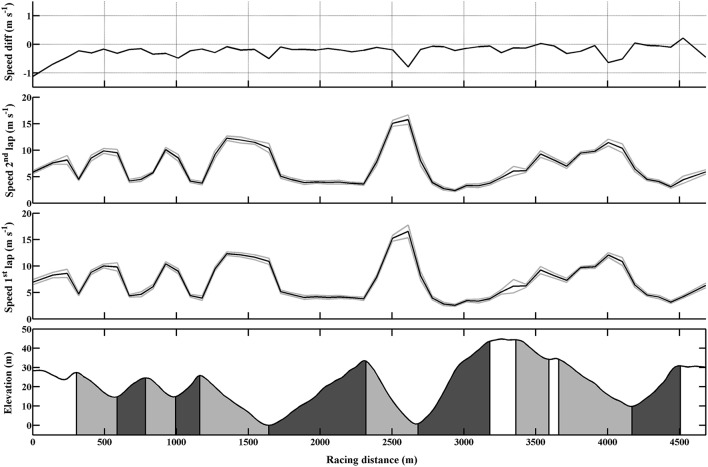
**Skiing speed (mean and standard deviation) during each lap and the mean speed differences between the two laps during the 10-km classical time-trial competition among 10 elite female cross-country skiers**. Note that the start and finish sections are not included in the comparison between laps.

The participants' ranking ranged from 1 to 39 in the competition. The relative time spent on the uphill, flat and downhill sections was 56, 16, and 28%, respectively (Table 2). The average speed during the race was 5.7 m·s^−1^, with a CV of 5.1%. The first lap was executed 30 ± 5 s faster than the second, with 18 ± 3, 6 ± 2, and 7 ± 2 of these seconds lost on uphill, flat, and downhill terrain, respectively (all *p* < 0.05). Time, speed and CV's of speed in each section are depicted in Figure 2; Table 2. Overall time-trial performance and the times spent on uphill, flat and downhill terrain during the race were positively correlated with distance FIS points (*r* = 0.92, 0.90, 0.78, 0.73, respectively, all *p* < 0.001).

The time spent on uphill (*r* = 0.98, *p* < 0.001) and flat (*r* = 0.91, *p* < 0.001) terrain during the race positively correlated with overall time-trial performance, and was more strongly correlated with overall performance than time spent on downhill terrain (*r* = 0.72, *p* < 0.019). Similar relationships were observed for uphill, flat, and downhill terrains within the specific sections and for both laps. The stepwise multiple regression model revealed that time spent on uphill, flat and downhill terrain explained 95.5% (*R*^2^ change = 0.955, std. β = 0.76, semi-partial *R*^2^ = 0.17), 0.3% (*R*^2^ change = 0.003, std. β = 0.14, semi-partial *R*^2^ = 0.00) and 4.2% (*R*^2^ change = 0.042, std. β = 0.18, semi-partial *R*^2^ = 0.01) of the total variance in 10-km performance, respectively (all IVs: *p* < 0.001).

The CVs for time spent within each section showed the highest variation in the uphill sections, followed by the flat sections (Table 2). Slower speed uphill on the second lap correlated with overall slower time for that lap (*r* = 0.90, *p* < 0.001).

Physiological capacities of the skiers are presented in Table 3. Correlations between performance-indices and physiological variables in the laboratory vs. 10-km time-trial performance and time spent in the different terrains are presented in Table 4. Distance covered during 3-min DP and DIA roller skiing, as well as body-mass normalized VO_2peak_ in both techniques were negatively correlated with overall time-trial performance (all *p* < 0.05). Distance covered during 3-min DP was negatively correlated with the time spent on flat and downhill terrain during the time-trial, and body-mass normalized VO_2peak_ in DP was negatively correlated with time spent on all types of terrain (all *p* < 0.05). In DIA, distance covered during the 3 min correlated negatively with time spent on uphill terrain (*p* < 0.05). Body-mass normalized VO_2peak_ in DIA was only significantly correlated with time spent on uphill terrain (*p* < 0.05). Body-mass and DP VO_2peak_ in L·min^−1^ correlated positively with the time spent downhill and overall time spent on the second 5-km lap, respectively (both *p* < 0.05).

**Table 3:** **Performance and physiological characteristics of the 10 elite female cross-country skiers during maximal and submaximal roller skiing in the double poling (DP) and diagonal stride (DIA) techniques (mean ± *SD*)**.

	**DP**	**DIA**
3-min performance (m)	945 ± 44	568 ± 27
VO_2peak_ (L·min^−1^)	3.79 ± 0.29	4.19 ± 0.31
VO_2peak_ (mL·min^−1^·kg^−1^)	61.1 ± 4.9	68.0 ± 4.6
%VO^#^_2max_	90 ± 4	100 ± 4
Peak heart rate (bpm)	182 ± 11	184 ± 8
% max heart rate^¤^	95 ± 3	97 ± 2
Peak blood lactate (mmol·L^−1^)	9.1 ± 1.4	10.6 ± 1.8
Submaximal O_2_-cost(L·min^−1^)	2.37 ± 0.27	2.92 ± 0.25
Submaximal O_2_-cost (mL·min^−1^·kg^−1^)	38.2 ± 2.3	47.3 ± 1.2
%VO^#^_2max_	63 ± 7	70 ± 4
Submaximal heart rate (bpm)	147 ± 23	157 ± 19
% max heart rate^¤^	76 ± 10	81 ± 8
Submaximal blood lactate (mmol·L^−1^)	1.9 ± 0.7	1.4 ± 0.7
Submaximal RPE (6–20)	11.6 ± 2.3	12.2 ± 2.4

*Submaximal tests were performed at 3% incline and 3.5 m·s^−1^ in DP and at 12% incline and 2 m·s^−1^ in DIA*.

# *Highest value achieved during the last 6 months using the same O_2_ analyzer, independent of technique or protocol*.

¤ *Highest value achieved during the last 6 months*.

**Table 4:** **The correlations (*r*-values) between laboratory measured physiological variables during maximal and submaximal roller skiing tests and overall 10-km time-trial (TT) performance and time spent in the different sections of terrain for the 10 elite female cross-country skiers**.

	**Overall TT (s)**	**Uphill terrain (s)**	**Flat terrain (s)**	**Downhill terrain (s)**
**DOUBLE POLING**				
3-min performance (m)	−0.67^*^	−0.55	−0.80^**^	−0.73^*^
VO_2peak_ (L·min^−1^)	−0.21	−0.04	−0.37	−0.69^*^
VO_2peak_ (mL·min^−1^·kg^−1^)	−0.68^*^	−0.63^*^	−0.69^*^	−0.68^*^
% VO_2max_	−0.10	−0.14	−0.29	−0.48
% max heart rate	0.84^**^	0.81^**^	0.73^*^	0.66^*^
Peak BLa (mmol·L^−1^)	0.48	0.47	0.47	0.30
Sub O2-cost (mL·min^−1^·kg^−1^)	0.40	0.31	0.63^*^	0.40
Sub %VO^#^_2max_	0.66^*^	0.78^*^	0.61	0.57
Sub % max heart rate^¤^	0.78^**^	0.75^*^	0.59	0.74^*^
Sub BLa (mmol·L^−1^)	0.86^**^	0.89^**^	0.78^**^	0.78^**^
Sub RPE (6–20)	0.82^**^	0.83^**^	0.69^*^	0.51
**DIAGONAL STRIDE**				
3-min performance (m)	−0.78^**^	−0.77^**^	−0.58	−0.62
VO_2peak_ (L·min^−1^)	−0.08	0.05	−0.14	−0.53
VO_2peak_ (mL·min^−1^·kg^−1^)	−0.66^*^	−0.63^*^	−0.48	−0.51
% VO_2max_	0.05	0.03	0.10	0.10
% max heart rate	0.09	0.18	−0.00	−0.25
Peak BLa (mmol·L^−1^)	0.10	0.03	0.28	0.25
Sub O2-cost (mL·min^−1^·kg^−1^)	0.43	0.58	0.27	0.16
Sub %VO^#^_2max_	0.60	0.67^*^	0.46	0.54
Sub % max heart rate^¤^	0.77^**^	0.78^**^	0.72^*^	0.59
Sub BLa (mmol·L^−1^)	0.58	0.78^**^	0.50	0.49
Sub RPE (6–20)	0.78^**^	0.82^**^	0.60	0.57

# *Highest value achieved during the last 6 months using the same O_2_ analyzer, independent of technique or protocol*.

¤ *Highest value achieved during the last 6 months*.

* *p <0.05*;

** *p <0.01*.

Oxygen cost in submaximal DP correlated positively with overall time-trial performance and the time spent on flat terrain (Table 4, both *p* < 0.05). Furthermore, the % of VO_2max_ utilized and blood lactate concentration in submaximal DP and %HR_max_ and RPE in both techniques correlated positively with overall time-trial performance (all *p* < 0.05).

## Discussion

The present study examined the contribution of performance on uphill, flat, and downhill sections to overall performance in an international 10-km classical time-trial in elite female cross-country skiers, as well as the relationships to physiological variables in the double poling and diagonal techniques. The main findings were as follows: (1) Performance on uphill and flat sections correlated most strongly with overall time-trial performance; (2) More than half of the race time was spent skiing uphill and ~95% of the variance in classical time-trial performance could be explained by performance during these sections; (3) All skiers utilized a positive pacing strategy where they decrease their speed in the second half of the race in all terrain sections, but no significant correlation between pacing strategy (i.e., changes in speed in the different terrains from the first to the second lap) and overall performance was found; (4) Distance covered during 3-min roller ski and body-mass normalized VO_2peak_ in both techniques correlated strongly with overall time-trial performance, with DP capacity tending to have the greatest impact on performance on flat and DIA capacity on uphill terrain.

### Analysis of the competition

Speed on uphill and flat sections of the race was most strongly associated with overall time-trial performance, and both correlations were significantly stronger than the impact of skiing fast on downhill terrain. In further support of this, the coefficients of variation within each section showed patterns similar to the correlations themselves, with the highest variation in time spent on the uphill sections, followed by the flat sections. Together with previous data from both sprint and distance skiing, these data indicate the presence of a hierarchy in the contribution of time spent on uphill, flat, and downhill terrains to overall time-trial performance (Andersson et al., 2010; Sandbakk et al., 2011; Bolger et al., 2015). In our case this pattern was observed consistently across all sections and on both laps among female elite skiers.

In further support of uphill skiing as the major contributor to overall time-trial performance, our stepwise multiple regression model showed that the time spent uphill had the highest *R*^2^ change and explained ~95% of the overall variance in performance. In the current study, this could be explained by the large amount of time spent uphill, together with the high between-athlete variability in uphill performance (i.e., the highest CVs were found uphill). Thus, only ~5% of the between-athlete variability in performance remained to be explained by the time spent on downhill and flat terrain. However, when uphill time is excluded from the model (i.e., when the semi-partial *R*^2^ for uphill terrain is subtracted from the overall *R*^2^ in the multiple regression model), the time spent downhill and on the flat together explain 83% of the total variability in race time. This is caused by high correlation between performance in the different terrains (i.e., high multi-collinearity between the IVs). Hence, in addition to the high importance of uphill skiing for overall race performance (Norman and Komi, 1987; Bergh and Forsberg, 1992; Mognoni et al., 2001; Andersson et al., 2010; Sandbakk et al., 2011; Bolger et al., 2015), our study reveals that the best performing female athletes are faster in all types of terrain.

As shown in previous studies on cross-country skiing, all skiers in our study utilized a positive pacing strategy with 2–4% decreases in speed over the second half of the race (Andersson et al., 2010; Bolger et al., 2015; Losnegard et al., 2016). While reductions in speed appeared in all terrain types in our study, most of the 30-s slower pace on the second lap of the race was lost uphill, a time loss that correlated with the overall slower pace on the second lap. However, no significant relationship between pacing strategy and overall performance was found, indicating that the skiers utilized relatively similar pacing strategies and that there are individual optimums in the magnitude of positive pacing. This is supported by a previous study done on female skiers (Losnegard et al., 2016), although other indications are that male skiers with higher endurance capacity (Andersson et al., 2010), or performance level (Losnegard et al., 2016) maintain speed better than those with a lower endurance capacity.

### Laboratory determinants of performance

As expected, endurance performance in the laboratory (i.e., distance covered on the 3-min test) and body-mass normalized VO_2peak_ in both DP and DIA techniques were strongly correlated with overall time-trial performance. These findings build upon previous research showing that female skiers at a world-class level perform better and exhibit higher VO_2peak_ both with the DP technique and with DIA than lower ranked counterparts (Sandbakk et al., 2016). Hence, the performance differences among female elite skiers can largely be explained by variations in production and utilization of aerobic energy in the specific techniques.

When correlated to specific terrain sections of the race, distance covered during 3-min DP in the laboratory was significantly associated with the time spent on flat and downhill terrain during the time-trial, whereas the body-mass normalized VO_2peak_ in DP was significantly correlated with time spent on all three terrains. This highlights the general importance of DP capacity in all types of terrain in today's cross-country skiing races (Sandbakk and Holmberg, 2014; Osteras et al., 2016), with DP capacity being particularly important for the flat terrain, where this technique is most commonly used.

In DIA, 3-min roller ski performance correlated most strongly with the time spent on uphill terrain, while body-mass normalized VO_2peak_ in DIA was only significantly correlated with the time spent uphill. This further demonstrates that performance indices and physiological capacity in the specific techniques influence on-snow performance in terrains where this technique is predominant during competitions. Overall, the high correlations between DP and DIA roller skiing capacity and performance on snow indicates that skiers of a high performance level should be tested in terrain-specific techniques in order to differentiate performance levels as accurately as possible. This could also be crucial for identification of an individual athlete's strengths and weaknesses and thereby have a practical relevance for their subsequent training strategy.

In downhill terrain the heavier skiers appeared to be faster, and in particular those with high aerobic power. The major portion of the positive effect of high body-mass on downhill performance may be explained by the greater horizontal component of gravitational forces acting on skiers. In addition, the ability to enter the downhill sections with higher speeds may profit those with additional high aerobic power.

As expected from their higher VO_2peak_, the submaximal roller skiing stages for both techniques tended to be less demanding for the fastest skiers during the time-trial on snow. In addition, the oxygen cost of DP correlated with the time spent on flat terrain, whereas oxygen cost in DIA showed a tendency to correlate with the time spent on uphill terrain. Although skiing efficiency in DP and DIA did not differ significantly between world-class and national-class female skiers (Sandbakk et al., 2016), we here show that oxygen cost of roller skiing may specifically relate to skiers' competitive speed in the same terrain during competition on snow. This applied particularly to DP where the “timing” required to produce power with short poling times is technically demanding (Lindinger et al., 2009). Thus, to achieve world-class performance, female skiers may profit from a well-developed skiing economy, in particular on flat terrain.

Although this study's results indicate that technique-specific VO_2peak_ rather than skiing efficiency/economy explains most of the performance variations on snow, the literature generally indicates a large in influence of efficiency on cross-country skiing performance (Sandbakk et al., 2010, 2013; Ainegren et al., 2013; Losnegard et al., 2013). For example, previous research showed that top-level cross-country skiers improved performance and work economy, but not VO_2max_, from the early preparation phase to the competition phase during a season (Losnegard et al., 2013). This is likely explained by the technical complexity of the sport, which is also the case in e.g., speed skating where a recent study suggested that 50% of the improvement in speed skating over the last 50 years is caused by more efficient athletes (even when suits, skates, and ice tracks are accounted for; Noordhof et al., 2016).

## Conclusions

Our present findings reveal that most time is spent uphill during an international 10-km classical time-trial for female cross-country skiers, and that the time spent uphill most strongly contributes to the overall race performance. However, high multi-collinearity between the performance on uphill, flat, and downhill terrains were present, indicating that the best performing athletes are generally faster in all types of terrain. Furthermore, all skiers utilized a positive pacing strategy, but no significant relationship between individual pacing strategies and overall performance was found.

The major portion of the performance differences could be explained by variations in VO_2peak_ in the specific techniques, highlighting the importance of technique-specific physiological capacity on performance in terrain where this technique is predominant. To perform at a world-class level in competitions, female skiers must exhibit both high peak oxygen uptake and well-developed skiing economy on both uphill and flat terrain.

## Author contributions

Conceived and designed the experiments: ØS, ET, JK, and AH. Performed the experiments: ØS, AH, JK, and KM. Analyzed the data: ØS, AH, TL, JK, and ØS. Wrote the paper: ØS, AH, JK, ET, TL, and ØS.

### Conflict of interest statement

The authors declare that the research was conducted in the absence of any commercial or financial relationships that could be construed as a potential conflict of interest.
